# Risk Stratification Using a Novel Nomogram for 2190 EGFR-Mutant NSCLC Patients Receiving the First or Second Generation EGFR-TKI

**DOI:** 10.3390/cancers14040977

**Published:** 2022-02-15

**Authors:** John Wen-Cheng Chang, Chen-Yang Huang, Yueh-Fu Fang, Ching-Fu Chang, Cheng-Ta Yang, Chih-Hsi Scott Kuo, Ping-Chih Hsu, Chiao-En Wu

**Affiliations:** 1 Division of Hematology-Oncology, Department of Internal Medicine, Linkou Chang Gung Memorial Hospital, College of Medicine, Chang Gung University, Taoyuan 333, Taiwan; wen1902@hotmail.com (J.W.-C.C.); b9202070@cgmh.org.tw (C.-Y.H.); mr0826@cgmh.org.tw (C.-F.C.); 2 Division of Thoracic Oncology, Department of Thoracic Medicine, Linkou Chang Gung Memorial Hospital, College of Medicine, Chang Gung University, Taoyuan 333, Taiwan; ma3859@cgmh.org.tw (Y.-F.F.); yang1946@cgmh.org.tw (C.-T.Y.); r5245@cgmh.org.tw (C.-H.S.K.); 8902049@cgmh.org.tw (P.-C.H.)

**Keywords:** non-small cell lung cancer, tyrosine kinase inhibitor, nomogram, prognostic factor

## Abstract

**Simple Summary:**

No comprehensive and simple prognostic model based on pretreatment factors exists for patients with epidermal growth factor receptor mutation-positive (EGFRm+) non-small cell lung cancer (NSCLC) undergoing EGFR-tyrosine kinase inhibitors (EGFR-TKIs). A total of 11 independent prognostic factors were identified by multivariate analysis, including performance status, morphology, mutation, stage, EGFR-TKIs, and metastasis to liver, brain, bone, pleura, adrenal gland, and distant lymph nodes. We established a nomogram based on independent pretreatment factors and used it to stratify EGFRm+ NSCLC patients undergoing EGFR-TKI treatment into five different risk groups for survival using recursive partitioning analysis. The performance of this nomogram was good and feasible, providing clinicians and patients with additional information for evaluating therapeutic options.

**Abstract:**

Epidermal growth factor receptor tyrosine kinase inhibitors (EGFR-TKIs) are the standard treatment for EGFR mutation-positive (EGFRm+) non-small cell lung cancer (NSCLC). This study aimed to create a novel nomogram to help physicians suggest the optimal treatment for patients with EGFRm+ NSCLC. Records of 2190 patients with EGFRm+ NSCLC cancer who were treated with EGFR-TKIs (including gefitinib, erlotinib, and afatinib) at the branches of a hospital group between 2011 and 2018 were retrospectively reviewed. Their clinicopathological characteristics, clinical tumor response, progression-free survival (PFS), and overall survival (OS) data were collected. Univariate and multivariate analyses were performed to identify potential prognostic factors to create a nomogram for risk stratification. Univariate analysis identified 14 prognostic factors, and multivariate analysis confirmed the pretreatment independent factors, including Eastern Cooperative Oncology Group performance status, morphology, mutation, stage, EGFR-TKIs (gefitinib, erlotinib, or afatinib), and metastasis to liver, brain, bone, pleura, adrenal gland, and distant lymph nodes. Based on these factors, a novel nomogram was created and used to stratify the patients into five different risk groups for PFS and OS using recursive partitioning analysis. This risk stratification can provide additional information to clinicians and patients when determining the optimal therapeutic options for EGFRm+ NSCLC.

## 1. Introduction

Although epidermal growth factor receptor tyrosine kinase inhibitors (EGFR-TKIs) have long been the standard treatment for EGFR mutation-positive (EGFRm+) non-small-cell lung cancer (NSCLC), the responses to EGFR-TKIs and the duration of treatment are variable. The median progression-free survival (PFS) for first-generation (1G: gefitinib and erlotinib) [1,2,3,4] and second-generation (2G: afatinib, dacomitinib) [5,6,7,8] TKIs is approximately 12 months. However, 20–30% of patients do not respond at all or only respond for a very short time (<3 months) [7,9], and the coexistence of multiple genetic, phenotypic, and functional factors may contribute to this intrinsic resistance [10].

Currently, frontline osimertinib [11,12], gefitinib plus chemotherapy [13], erlotinib plus bevacizumab [14], and erlotinib plus ramucirumab [15] are new treatment strategies due to demonstrated improvements in PFS and overall survival (OS) based on phase III studies. Due to considerations regarding cost-effectiveness and the toxicities associated with these regimens, 1G/2G TKI monotherapy remains widely used in clinical practice, whereas these other regimens may serve as alternative options for EGFRm+ NSCLC patients. Therefore, the rapid and accurate identification of patients with a high risk of progression who may benefit from more aggressive treatment represents a currently unmet need in daily oncological practice.

Only a few molecular studies examining mutations in TP53 [16] and KRAS [17] have reported the negative influence of EGFR-TKIs efficacy. Aside from these molecular studies, few studies utilizing simple models were currently available for clinicians and patients. Therefore, we aimed to develop a simple and easily assessed prognostic model using a nomogram analysis based on the clinical features of EGFRm+ NSCLC patients.

## 2. Materials and Methods

### 2.1. Patients and Data Collection

All patients’ data were obtained from the Cancer Registry System using the Chang Gung Research Database [18], and comprehensive medical records were obtained from all branches of a hospital group in Taiwan [19]. The records of patients diagnosed with advanced lung cancer (stage IIIB or IV based on AJCC, 7th edition) in the Cancer Registry System with documented Eastern Cooperative Oncology Group (ECOG) performance status (PS), common EGFR mutations (exon 19 deletion and L858R mutation) who were treated with first-line EGFR-TKIs (gefitinib, erlotinib, and afatinib) from January 2011 to January 2018 were retrospectively reviewed. This study aimed to examine patients treated with EGFR-TKI monotherapy for first-line systemic treatment; therefore, patients treated with concurrent chemotherapy, concurrent bevacizumab, second-line systemic treatment, or neoadjuvant treatments were excluded. Patients with active cancer were also excluded. Finally, a total of 2190 EGFRm+ NSCLC patients treated with 1G/2G EGFR-TKIs as first-line treatment were analyzed in this study, including 1059 patients treated with gefitinib, 496 with erlotinib, and 635 with afatinib.

The clinical characteristics of the 2190 patients who received EGFR-TKIs as first-line treatments were retrospectively reviewed. The clinicopathological features, including age, sex, smoking history, ECOG PS score, stage, TKI use, tumor morphology, tumor involvement, EGFR mutation (exon 19 deletion, L858R), and tumor response were collected. The last follow-up time point assessed by this study was May 2021.

This study was approved by the Institutional Review Board of CGMH (201901395B0C501). Patients’ informed consent was not required due to the retrospective nature of this study.

### 2.2. Treatment and Response Evaluation

The EGFRm+ NSCLC patients were treated with 1G/2G EGFR-TKI monotherapy until disease progression or intolerable toxicity. Physicians adjusted the dose and schedule of EGFR-TKIs based on the patients’ clinical conditions and the occurrence of adverse events. The tumor response was evaluated by chest radiography, computed tomography (CT), or positron emission tomography (PET) and determined according to the Response Evaluation Criteria in Solid Tumors 1.1 (RECIST 1.1). The best clinical tumor response was recorded as complete response (CR), partial response (PR), stable disease (SD), or progressive disease (PD). Any tumor response that was not assessed before death or discontinuation for any reason was recorded as “not assessed (NA)”. The objective response rate (ORR) was defined as the proportion of patients with CR and PR. The disease control rate (DCR) was defined as the proportion of the patients with CR, PR, and SD. PFS was defined as the duration from the first day of EGFR-TKI treatment until the first radiological evidence of disease progression, the last dose of EGFR-TKI, death, or the most recent follow-up timepoint. Patients who experienced no progression and no death during treatment were censored from the PFS analysis. The study aim was to evaluate EGFR-TKI monotherapy; therefore, all patients treated with additional anti-tumor agents, such as chemotherapy or bevacizumab, after the initiation of TKI treatment, were censored at the start of combination treatment. Progression was defined when patients who received no sequential treatment experienced radiological progression or death within one month after EGFR-TKI discontinuation. OS was defined as the duration from the first day of afatinib treatment until the date of death or last follow-up. The data for patients who did not experience death were censored during survival curve analysis.

### 2.3. Statistical Analysis

Continuous variables were compared using the ANOVA test. Categorical variables were compared using Pearson’s Chi-square test or Fisher’s exact test, based on expected values. Survival was assessed using the Kaplan-Meier curve, and the log-rank test was applied to perform comparisons between groups. Univariate and multivariate analyses were conducted to investigate the multivariable relationships between predictors and PFS and to identify independent factors. Cox regression analysis was used for multivariate analyses and to formulate the nomogram.

### 2.4. Nomogram Creation and Statistical Software

A nomogram was analyzed by R software (R version 4.0.5, R Core Team, 2021, R Foundation for Statistical Computing, Vienna, Austria) with the rms package and other dependent packages. The concordance index (C-index) was applied to measure the performance of the nomogram. A calibration curve was plotted by comparing the nomogram-predicted versus observed probability of survival. For internal validation, bootstrapping with 1000 resamples was used.

To analyze the usefulness of the nomogram score as a prognostic factor for progression, we used recursive partitioning analysis (RPA), a statistical methodology that creates a survival analysis tree to establish an optimal cutoff point that better predicts progression [20]. SPSS (IBM Corp. Released 2015. IBM SPSS Statistics for Windows, Version 23.0. IBM Corp., Armonk, NY, USA) was used for statistical analyses. A *p*-value ≤ 0.05 was considered significant.

## 3. Results

### 3.1. Patient Characteristics

A total of 2190 EGFRm+ and treatment-naïve NSCLC patients undergoing EGFR-TKI monotherapy were included in the current study. The assessment of clinical tumor response showed that 9 and 1452 patients achieved CR (0.4%) and PR (66.3%), respectively, resulting in an ORR of 66.7%. In addition, 324 (14.8%) patients had SD, leading to a DCR of 81.5%. However, 189 (8.6%) patients had PD, and 216 (9.9%) patients were categorized as NA.

The mean age was 67.0 years (standard deviation: 12.1 years). The responders were significantly younger than patients who did not respond. Patients aged ≤65 years achieved a higher response rate than patients aged >65 years. No difference in tumor response was observed between male and female patients. The majority (78%) of patients had an ECOG PS of 0–1. Patients with good PS had a higher response rate than patients with poor PS (PS of 2–4). Three-quarters (74.2%) of patients were categorized as never-smokers, and smoking status was not associated with tumor response.

Most patients had adenocarcinoma (98.2%) and were classified as stage IV (93.2%). Among the EGFR mutation, the L858R (52.8%) mutation was slightly more common than the exon 19 deletion (47.2%). As expected, patients with adenocarcinoma and exon 19 deletion had a significantly higher ORR than patients with non-adenocarcinoma and L858R mutation. The clinical stage was not significantly associated with tumor response.

Bone (46.2%) was the most common metastatic site, followed by pleura (45.0%), lung (39.1%), brain (29.7), and liver (13.2%). Metastasis to the lung, liver, brain, bone, and distant lymph nodes was significantly associated with tumor response. Except for lung metastasis, other metastases were negatively associated with tumor response. All baseline characteristics according to tumor response are summarized in Table 1.

### 3.2. Influence of Clinical Variables on PFS

Univariate analysis was performed to identify possible prognostic factors (*p* < 0.1), including PS (0 vs. 1/2 vs. 3/4, *p* < 0.0001), morphology (adenocarcinoma vs. non-adenocarcinoma, *p* = 0.001), mutation (L858R vs. exon 19 deletion, *p* = 0.055), stage (IIIB vs. IV, *p* < 0.0001), EGFR-TKIs (*p* < 0.0001), lung metastasis (*p* = 0.032), liver metastasis (*p* < 0.0001), brain metastasis (*p* < 0.0001), bone metastasis (*p* < 0.0001), pleural metastasis (*p* < 0.0001), adrenal metastasis (*p* < 0.0001), distant lymph node metastasis (*p* = 0.017), pericardial metastasis (*p* < 0.0001), and clinical tumor response (*p* < 0.0001; Table 2).

The pretreatment variables with a *p*-value less than 0.1 in the univariate analysis were included in the regression formula. Clinical tumor response was not included in the regression because this factor cannot be assessed before treatment. Using a multivariate Cox regression model, 11 variables were selected as independent prognostic factors, PS (PS 1/2 vs. PS 0, adjusted hazard ratio [AHR]: 1.271, 95% confidence interval [CI]: 1.117–1.446, *p* < 0.001; PS 3/4 vs. PS 0, AHR: 1.627, 95% CI: 1.326–1.997, *p* < 0.001); morphology (non-adenocarcinoma vs. adenocarcinoma, AHR: 1.614, 95% CI: 1.132–2.301, *p* = 0.010); EGFR mutation (L858R vs. exon 19 deletion, AHR: 1.099, 95% CI: 1.002–1.206, *p* = 0.045); stage (IV vs. IIIB, AHR: 1.454, 95% CI: 1.179–1.793, *p* < 0.001), TKI use (erlotinib vs. afatinib, AHR: 1.274, 95% CI: 1.117–1.454, *p* < 0.001; gefitinib vs. afatinib, AHR: 1.461, 95% CI: 1.307–1.633, *p* < 0.0001); lung metastasis (AHR: 1.467, 95% CI: 1.276–1.687, *p* < 0.0001), brain metastasis (AHR: 1.222, 95% CI: 1.099–1.360, *p* < 0.001); bone metastasis (AHR: 1.328, 95% CI: 1.204–1.465, *p* < 0.0001); pleural metastasis (AHR: 1.360, 95% CI: 1.232–1.500, *p* < 0.0001); adrenal metastasis (AHR: 1.283, 95% CI: 1.085–1.516, *p* = 0.004); and distant lymph node metastasis (AHR: 1.175, 95% CI: 1.008–1.371, *p* = 0.040; Table 2).

### 3.3. Establishment of a Prognostic Nomogram Based on Pretreatment Variables

**Figure 1:**
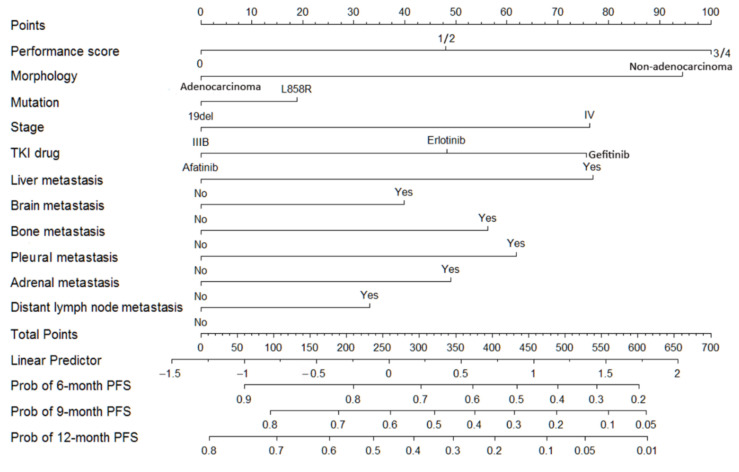
Nomogram based on the probability of progression-free survival (PFS) using the Cox regression model from 2190 EGFR mutation-positive non-small cell lung cancer (NSCLC) patients.

**Figure 2:**
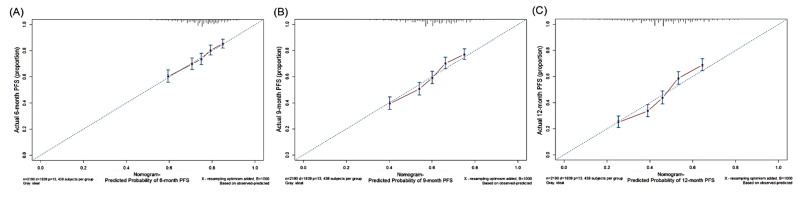
Calibration curves showing nomogram-predicted (**A**) 6-month, (**B**) 9-month, and (**C**) 12-month progression-free survival (PFS) and actually observed survival.

To establish a prognostic nomogram, 11 variables with *p* < 0.05 identified by the multivariate Cox regression analysis were selected (Figure 1). The C-index for the model was 0.626 (95% CI: 0.612–0.640) when comparing nomogram-predicted outcomes against actual observed outcomes. The calibration curves for the probability of survival at 6, 9, and 12 months after EGFR-TKI use revealed a good agreement between the nomogram prediction and actual observation (Figure 2). The formula (Table 3) included PS (PS0: 0 points, PS 1/2: 48 points, PS 3/4: 100 points), morphology (adenocarcinoma: 0 points, non-adenocarcinoma: 94 points), EGFR mutation (exon 19 deletion: 0 points, L858R: 19 points), stage (IIIB: 0 points, IV: 76 points), TKI use (afatinib: 0 points, erlotinib: 48 points, gefitinib: 76 points), lung metastasis (77 points), brain metastasis (40 points), bone metastasis (56 points), pleural metastasis (62 points), adrenal metastasis (49 points), and distant lymph node metastasis (33 points).

A logistic regression model was derived to predict 6-, 9-, and 12-month PFS, based on the total points determined by the nomogram (Table 4, Figure 1 and Figure 2). Higher values corresponded to a higher estimated risk of progression.

### 3.4. Risk Stratification by the Nomogram

**Figure 3:**
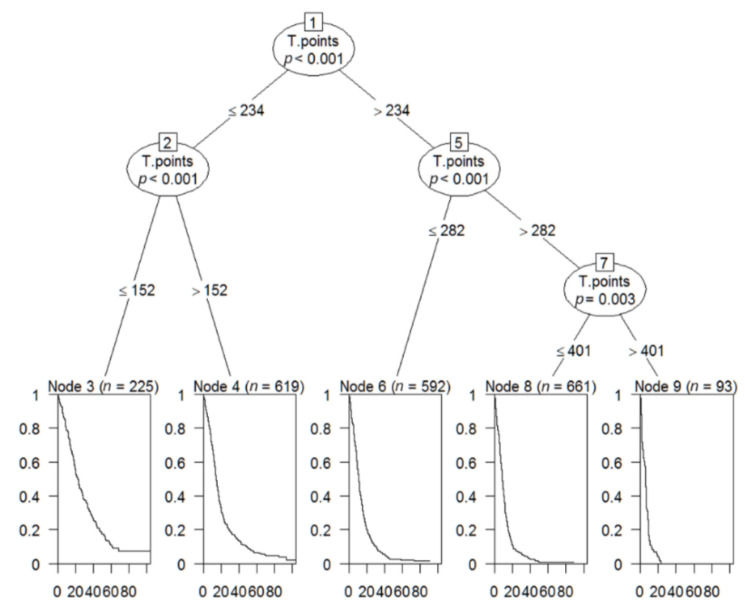
A survival analysis tree was used to establish an optimal cutoff point to better predict tumor progression.

**Figure 4:**
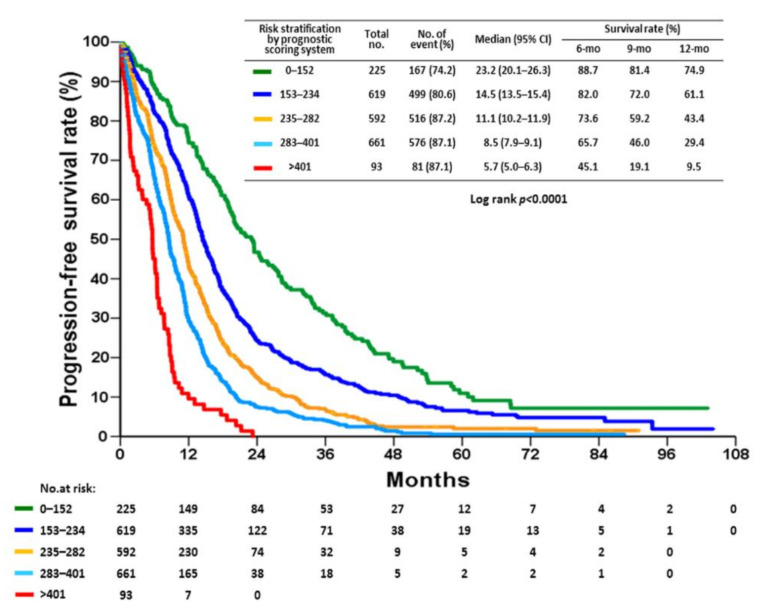
Kaplan-Meier plot of progression-free survival for 2190 patients according to risk stratification based on nomogram points.

RPA was used to analyze the usefulness of the nomogram score as a prognostic factor for progression (Figure 3). Based on the RPA results, this cohort was divided into five risk groups based on nomogram scores. A total of 93 patients were categorized as the highest-risk group (total points: >401, median PFS: 5.7 months), 611 patients as the high-risk group (total points: 283–401, median PFS: 8.5 months), 592 patients as the intermediate-risk group (total points: 235–282, median PFS: 11.1 months), 619 patients as the low-risk group (total points: 153–234, median PFS: 14.5 months), and 152 patients as the lowest-risk group (total points: 0–152, median PFS: 23.3 months; Figure 4). Although OS was not the primary endpoint examined in the current study, this risk stratification approach was also able to predict the risk for OS (Supplementary Figure S1).

## 4. Discussion

In the current study, a novel nomogram based on pretreatment and easily assessed clinical factors was developed to predict PFS among EGFRm+ NSCLC patients. Overall, 11 pretreatment factors, including PS, stage, EGFR mutation, morphology, TKI use, and tumor metastases were identified. This nomogram can be used to predict the possibility of 6-, 9-, and 12-month PFS and stratify patients into different risk groups for PFS and OS. This nomogram can be easily assessed, allowing patients and clinicians to better evaluate optimal therapeutic options before the initiation of TKIs.

EGFR-TKIs have long been the standard treatment for EGFRm+ NSCLC, and sequential 1G/2G EGFR-TKIs, followed by osimertinib, may provide better survival outcomes than frontline osimertinib [21]. The critical point at which sequential treatment becomes necessary is the occurrence of the acquired T790M resistance mutation. Generally, pretreatment of individuals harboring the exon 19 deletion is associated with a higher risk of developing the acquired T790M resistance mutation than pretreatment of individuals harboring the L858 mutation [22]. In addition, the clinical tumor response [23] and a longer duration of EGFR-TKI treatment (≥12 months) [24,25,26,27] were reported to be associated with a higher frequency of T790M mutation acquisition. Therefore, patients in the low-risk groups with predicted PFS ≥12 months may be presumed to have a higher chance of acquiring the T790M mutation and are suitable for sequential osimertinib use, although acquired T790M and sequential osimertinib were not evaluated in the current study. In contrast, the patients in the higher-risk groups may consider other therapeutic options, such as frontline osimertinib [11,12] or the addition of chemotherapy [13], bevacizumab [14], or ramucirumab [15].

In this model, tumor stage, mutation status, PS, and metastatic sites were identified as prognostic factors, and these factors are well-known prognostic factors for NSCLC. Liver metastasis is an important prognostic factor for NSCLC patients treated with EGFR-TKIs [28,29]. In the current study, we found that the different metastatic sites may have distinct prognostic values. Liver metastasis was associated with the highest score of 77 points, followed by metastasis to the pleura (62 points), bone (56 points), adrenal gland (49 points), brain (40 points), and distant lymph nodes (33 points). Although lung and pericardial metastases were identified as prognostic factors in univariate analysis, they were not independent prognostic factors in the current cohort on multivariate analysis. Peritoneal metastasis is a rare metastatic site with a poor prognosis [30,31], and only six patients were identified in the current study. The median PFS of patients with peritoneal metastasis was 3.9 months, and no significant effect of this metastatic site was found in the univariate analysis due to the limited number of cases. The occurrence of multiple metastases likely increases both intra- and intertumoral heterogeneity [32,33]; however, we only assessed the genetic status of one tumor and presumed that all tumors shared the same genetic alterations. Therefore, the mixed response to EGFR-TKIs indicates increased heterogeneity, which is associated with a higher risk for progression in patients with metastasis to multiple organs.

The morphology is one prognostic factor in the current study. Although EGFR mutation has been reported in non-adenocarcinoma lung cancer, particularly in squamous cell carcinoma (SCC), the PFS of EGFR-TKIs for EGFR mutated lung SCC was shorter than the patients with EGFR mutated adenocarcinoma [34]. Mutations in other genes have been reported as the potential understanding mechanisms of resistance to EGFR-TKI in lung SCC [34].

**Figure 5:**
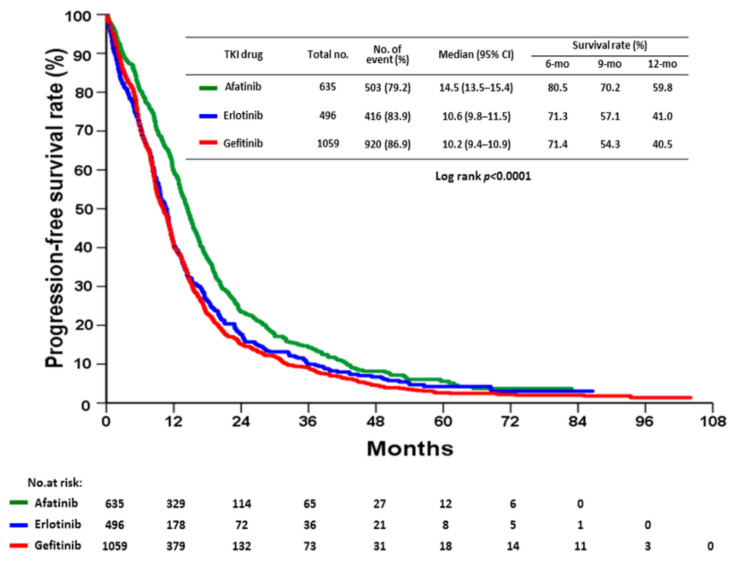
Kaplan–Meier plot of progression-free survival for 2190 patients according to the use of different EGKI-TKIs.

The only modifiable factor identified in the current study was TKI use. The LUX-Lung 7 study was the only prospective study to compare afatinib with gefitinib [7]. Although the median PFS values were 11.0 and 10.9 months for afatinib and gefitinib, respectively, the HR was 0.73 (95% CI: 0.57–0.95, *p* = 0.017), indicating that afatinib significantly improved PFS in this trial. Real-world studies have all demonstrated that patients undergoing afatinib had better PFS than patients undergoing 1G TKIs [26,35,36,37,38]. Consistent with previous real-world experience, afatinib demonstrated the best outcomes among the 3 EGFR-TKIs examined (Table 2 and Table 3, Figure 5 and Supplementary Figure S2). Therefore, the selection of EGFR-TKIs should depend not only on the patients’ tolerability but also on the risk stratification for tumor progression.

Only a few studies have attempted to develop a prognostic model like a nomogram. In 2014, Keam et al. developed a nomogram based on disease status (recurrent and metastatic), PS, line of TKI, response to EGFR-TKIs, and bone metastasis [39] based on an analysis of 306 patients undergoing TKI therapy. In contrast to the model developed by Keam et al., we only enrolled treatment-naïve patients and did not include the tumor response. Although tumor response is commonly identified as among the most important prognostic factors, tumor response cannot be assessed prior to treatment. Another study enrolled 129 patients with only brain metastasis, and most of the identified factors were associated with brain metastasis, including the number of brain tumors and the interval from diagnosis to brain metastasis, which were not included in the current study [40]. Recently, some novel studies have incorporated the results of 18F-fluorodeoxyglucose PET/CT analyses [41] or CT-based radiomics [42] to create nomograms for the prediction of survival in EGFRm+ NSCLC patients; however, these require an experienced radiologist, which may increase the difficulty in real-world settings. The current study aimed to create a simple, objective, easily assessed nomogram based on clinical factors that are readily available in daily practice.

Although osimertinib has not been analyzed in the current study, the prognostic factors of osimertinib may be similar to the factors of 1G/2G EGFR-TKIs. In a retrospective study of 538 patients undergoing first-line osimertinib, sex, stage, malignant pleural effusion, liver metastasis, mutation type and programmed cell death-ligand 1 (PD-L1) expression were associated with PFS by multivariate analysis [43]. Most of the prognostic factors for osimertinib treatment were consistent with the factors identified in the current study. As osimertinib demonstrated much longer PFS than 1G EGFR-TKIs in the FLAURA study [11,12], a nomogram specific for osimertinib is warranted.

Bias may exist in the current study due to its retrospective nature. However, most of the variables we selected for analysis are objective rather than subjective variables, which might minimize bias. All of the studied EGFR-TKIs are reimbursed by the Taiwan National Health Insurance program, and clinicians should provide evidence of non-PD for the continuation of EGFR-TKI therapy. In addition, OS was not used as the primary endpoint of the current study because OS can be heavily influenced by subsequent treatments. This study enrolled patients treated with TKIs starting in 2011; however, the overall therapeutic strategy has undergone various changes over the past decade. We also evaluated the prognostic abilities of our nomogram on OS as a separate analysis, and a significant difference was found according to risk stratification, which suggests that this model could also be used to estimate OS (Supplementary Figure S1). Furthermore, the current study only enrolled patients bearing tumors harboring common EGFR mutations (exon 19 deletion and L858R). Due to the heterogeneity of uncommon mutations and their distinct responses to 1G/2G EGFR-TKIs, uncommon mutations should be excluded when assessing patients to prevent interference from other factors during the application of this model [44,45,46]. The last but not the least limitation in the current study is that osimertinib, an important 3G EGFR-TKI available in clinical practice, was not included. No prospective study comparing osimertinib with 2G EGFR-TKIs is available. The only retrospective study demonstrated that osimertinib and afatinib showed similar OS [47]. Because it has a high price and offers no survival benefit in an Asian subgroup [11], osimertinib is not fully reimbursed in Asian countries, including Taiwan. 1G/2G EGFR-TKIs are still the major TKIs in clinical practice in most Asian countries. Therefore, the nomogram is still valuable for most clinicians and patients.

## 5. Conclusions

In the current study, a novel nomogram based on pretreatment clinical factors was developed that was able to stratify EGFRm+ NSCLC patients undergoing 1G/2G TKI monotherapy into five different risk groups. This risk stratification can provide additional information to clinicians and patients when determining the optimal therapeutic options for EGFRm+ NSCLC.

## Figures and Tables

**Table 1:** Characteristics based on clinical tumor response.

Characteristics	Total (*n* = 2190)	Response			*p*-Value
		CR/PR (*n* = 1461)	SD (*n* = 324)	PD/NA (*n* = 405)	
Basic data					
Age (years), mean ± SD	67.0 ± 12.1	66.1 ± 12.0	67.5 ± 12.2	69.8 ± 12.0	<0.0001
≤65	989 (45.2)	695 (70.3)	144 (14.6)	150 (15.1)	0.001
>65	1201 (54.8)	766 (63.8)	180 (15.0)	255 (21.2)	
Sex					
Male	1321 (60.3)	896 (67.8)	186 (14.1)	239 (18.1)	0.357
Female	869 (39.7)	565 (65.0)	138 (15.9)	166 (19.1)	
Performance score					
0	360 (16.4)	262 (72.8)	53 (14.7)	45 (12.5)	<0.0001
1	1350 (61.6)	914 (67.7)	221 (16.4)	215 (15.9)	
2	289 (13.2)	181 (62.6)	28 (9.7)	80 (27.7)	
3	130 (5.9)	70 (53.8)	17 (13.1)	43 (33.1)	
4	61 (2.8)	34 (55.7)	5 (8.2)	22 (36.1)	
Smoking					
Yes	508 (23.2)	328 (64.6)	79 (15.5)	101 (19.9)	0.831
No	1625 (74.2)	1095 (67.4)	236 (14.5)	294 (18.1)	
Unknown	57 (2.6)	38 (66.7)	9 (15.8)	10 (17.5)	
Tumor characteristics					
Morphology					
Adenocarcinoma	2151 (98.2)	1443 (67.1)	318 (14.8)	390 (18.1)	0.004
Non-adenocarcinoma	39 (1.8)	18 (46.2)	6 (15.3)	15 (38.5)	
Mutation					
Exon 19 deletion	1034 (47.2)	724 (70.1)	138 (13.3)	172 (16.6)	0.008
L858R	1156 (52.8)	737 (63.8)	186 (16.0)	233 (20.2)	
Stage					
IIIB	149 (6.8)	109 (73.2)	17 (11.4)	23 (15.4)	0.219
IV	2041 (93.2)	1352 (66.3)	307 (15.0)	382 (18.7)	
TKI therapy					
Drug					
Afatinib	635 (29.0)	454 (71.5)	89 (14.0)	92 (14.5)	0.005
Erlotinib	496 (22.6)	313 (63.1)	70 (14.1)	113 (22.8)	
Gefitinib	1059 (48.4)	694 (65.5)	165 (15.6)	200 (18.9)	
Metastatic site					
Lung					
Yes	857 (39.1)	605 (70.6)	113 (13.2)	139 (16.2)	0.008
No	1333 (60.9)	856 (64.2)	211 (15.8)	266 (20.0)	
Liver					
Yes	290 (13.2)	187 (64.5)	31 (10.7)	72 (24.8)	0.004
No	1900 (86.8)	1274 (67.1)	293 (15.4)	333 (17.5)	
Brain					
Yes	650 (29.7)	433 (66.7)	77 (11.8)	140 (21.5)	0.007
No	1540 (70.3)	1028 (66.8)	247 (16.0)	265 (17.2)	
Bone					
Yes	1012 (46.2)	651 (64.3)	150 (14.8)	211 (20.8)	0.027
No	1178 (53.8)	810 (68.8)	174 (14.7)	194 (16.5)	
Pleura					
Yes	986 (45.0)	639 (64.8)	153 (15.5)	194 (19.7)	0.227
No	1204 (55.0)	822 (68.3)	171 (14.2)	211 (17.5)	
Adrenal					
Yes	189 (8.6)	117 (61.9)	25 (13.2)	47 (24.9)	0.061
No	2001 (91.4)	1344 (67.2)	299 (14.9)	358 (17.9)	
Distant lymph node					
Yes	223 (10.2)	144 (64.6)	25 (11.2)	54 (24.2)	0.035
No	1967 (89.8)	1317 (67.0)	299 (15.2)	351 (17.8)	
Pericardium					
Yes	45 (2.1)	27 (60.0)	6 (13.3)	12 (26.7)	0.361
No	2145 (97.9)	1434 (66.9)	318 (14.8)	393 (18.3)	
Peritoneum					
Yes	6 (0.3)	4 (66.7)	0	2 (33.3)	0.448
No	2184 (99.7)	1457 (66.7)	321 (14.8)	403 (18.5)	

Notes: Data are reported as number (percentage) unless otherwise stated. Continuous variables were compared using an ANOVA test. Categorical variables were compared using Pearson’s Chi-square test or Fisher’s exact test, based on expected values. Abbreviations: TKI, tyrosine kinase inhibitor; CR, complete response; PR, partial response; SD, stable disease; PD, progressive disease; NA, not assessed.

**Table 2:** Univariate and multivariate analysis of prognostic factors for progression-free survival (PFS).

Parameters	Total *n*	*n* of Events (%)	Median (Months)	95% CI	*p*-Value	Hazard Ratio	95% CI	*p*-Value
Age (years)								
≤65	989	873 (88.3)	11.4	10.8–11.9	0.289	-		
>65	1201	966 (80.4)	11.4	10.6–12.2				
Sex								
Male	869	749 (86.2)	11.1	10.3–11.8	0.198	-		
Female	1321	1090 (82.5)	11.5	10.9–12.2				
Performance score						-		
0	360	284 (78.9)	14.4	12.8–16.0	<0.0001	1	-	-
1/2	1639	1408 (85.9)	11.3	10.8–11.8		1.271	1.117–1.446	<0.001
3/4	191	147 (77.0)	6.4	5.3–7.6		1.627	1.326–1.997	<0.0001
Smoking								
Yes	508	437 (86.0)	11.0	10.0–12.1	0.275	-		
No	1625	1351 (83.1)	11.5	10.9–12.1				
Unknown	57	51 (89.5)	10.3	7.7–12.9				
Morphology								
Adenocarcinoma	2151	1807 (84.0)	11.5	11.0–12.0	0.001	1	-	-
Non-adenocarcinoma	39	32 (82.1)	5.3	4.4–6.2		1.614	1.132–2.301	0.010
Mutation								
Exon 19 deletion	1034	884 (85.5)	11.9	11.2–12.6	0.055	1	-	-
L858R	1156	955 (82.6)	10.9	10.2–11.5		1.099	1.002–1.206	0.045
Stage								
IIIB	149	112 (75.2)	21.8	18.0–25.6	<0.0001	1	-	-
IV	2041	1727 (84.6)	11.1	10.6–11.6		1.454	1.179–1.793	<0.001
TKI drug								
Afatinib	635	503 (79.2)	14.5	13.5–15.4	<0.0001	1	-	-
Erlotinib	496	416 (83.9)	10.6	9.8–11.5		1.274	1.117–1.454	<0.001
Gefitinib	1059	920 (86.9)	10.2	9.4–10.9		1.461	1.307–1.633	<0.0001
Lung metastasis								
Yes	857	725 (84.6)	11.1	10.4–11.9	0.032	1.029	0.935–1.132	0.561
No	1333	1114 (83.6)	11.5	10.8–12.2		1	-	-
Liver metastasis								
Yes	290	255 (87.9)	8.5	7.8–9.3	<0.0001	1.467	1.276–1.687	<0.0001
No	1900	1584 (83.4)	11.8	11.2–12.3		1	-	-
Brain metastasis								
Yes	650	546 (84.0)	9.4	8.6–10.3	<0.0001	1.222	1.099–1.360	<0.001
No	1540	1293 (84.0)	11.9	11.3–12.5		1	-	-
Bone metastasis								
Yes	1012	871 (82.2)	9.9	9.2–10.6	<0.0001	1.328	1.204–1.465	<0.0001
No	1178	968 (86.1)	12.9	12.1–13.7		1	-	-
Pleural metastasis								
Yes	986	848 (86.0)	10.8	10.1–11.5	<0.0001	1.360	1.232–1.500	<0.0001
No	1204	991 (82.3)	12.4	11.5–13.2		1	-	-
Adrenal metastasis								
Yes	189	26 (86.2)	8.2	7.0–9.4	<0.0001	1.283	1.085–1.516	0.004
No	2001	1676 (83.8)	11.6	11.1–12.1		1	-	-
Distant LN metastasis								
Yes	223	187 (83.9)	8.7	6.9–10.5	0.017	1.175	1.008–1.371	0.040
No	1967	1652 (84.0)	11.5	11.0–12.0		1	-	-
Pericardial metastasis								
Yes	45	39 (86.7)	7.6	4.3–10.8	0.001	1.221	0.882–1.690	0.229
No	2145	1800 (83.9)	11.5	11.0–12.0		1	-	-
Peritoneal metastasis								
Yes	6	6 (100.0)	3.9	0.1–13.2	0.122	-		
No	2184	1833 (83.9)	11.4	10.9–11.9				
Tumor Response								
CR/PR	1461	1252 (85.7)	13.4	12.8–14.1	<0.0001	-		
SD	324	271 (83.6)	11.6	10.0–13.2				
PD/NA	405	316 (88.0)	1.9	1.7–2.1				

Abbreviations: CI, confidence interval; TKI, tyrosine kinase inhibitor; LN, lymph node; CR, complete response; PR, partial response; SD, stable disease; PD, progressive disease; NA, not assessed.

**Table 3:** Multivariate analysis of prognostic variables associated with progression-free survival (PFS).

Prognostic Variables	Hazard Ratio	95% CI		*p*-Value	Points Assigned in Nomogram
		Lower	Upper		
Performance score					
0	1	-	-	-	0
1/2	1.274	1.120	1.449	<0.001	48
3/4	1.655	1.351	2.027	<0.001	100
Morphology					
Adenocarcinoma	1	-	-	-	0
Non-adenocarcinoma	1.610	1.129	2.294	0.008	94
Mutation					
Exon 19 deletion	1	-	-	-	0
L858R	1.100	1.003	1.206	0.044	19
Stage					
IIIB	1	-	-	-	0
IV	1.468	1.195	1.805	<0.001	76
TKI drug					
Afatinib	1				0
Erlotinib	1.276	1.118	1.455	<0.001	48
Gefitinib	1.464	1.310	1.636	<0.0001	76
Liver metastasis					
Yes	1.474	1.282	1.694	<0.0001	77
No	1	-	-	-	0
Brain metastasis					
Yes	1.223	1.099	1.360	<0.001	40
No	1	-	-	-	0
Bone metastasis					
Yes	1.328	1.204	1.464	<0.0001	56
No	1	-	-	-	0
Pleural metastasis					
Yes	1.366	1.239	1.506	<0.0001	62
No	1	-	-	-	0
Adrenal metastasis					
Yes	1.281	1.084	1.513	0.004	49
No	1	-	-	-	0
Distant LN metastasis					
Yes	1.182	1.014	1.378	0.033	33
No	1	-	-	-	0

Abbreviations: CI, confidence interval; TKI, tyrosine kinase inhibitor; LN, lymph node.

**Table 4:** Prognostic scoring system.

**Nomogram Points**	**Probability of 6-Month PFS**
602	0.20
544	0.30
490	0.40
434	0.50
374	0.60
303	0.70
209	0.80
60	0.90
**Nomogram Points**	**Probability of 9-Month PFS**
612	0.05
559	0.10
488	0.20
431	0.30
376	0.40
321	0.50
260	0.60
189	0.70
96	0.80
**Nomogram Points**	**Probability of 12-Month PFS**
613	0.01
527	0.05
475	0.10
404	0.20
348	0.30
292	0.40
237	0.50
176	0.60
105	0.70
12	0.80

Abbreviations: PFS, progression-free survival.

## Data Availability

The data presented in this study are available on request from the corresponding author.

## Supplementary Materials

The following supporting information can be downloaded at: https://www.mdpi.com/article/10.3390/cancers14040977/s1, Figure S1: Kaplan-Meier plot of overall survival for 2190 patients according to risk stratification based on nomogram points, Figure S2: Kaplan-Meier plot of overall survival for 2190 patients according to the use of different EGKI-TKIs.
