# Supplementary material for: Risk Stratification Using a Novel Nomogram for 2190 EGFR-Mutant NSCLC Patients Receiving the First or Second Generation EGFR-TKI
Source: Cancers (Basel). 2022 Feb 15;14(4):977. doi: 10.3390/cancers14040977 (PMC8870328; doi:10.3390/cancers14040977)
Supplement: Supplementary file 1 [file cancers-14-00977-s001.zip › cancers-1568134-supplementary.pdf]

Supplementary Materials

Risk Stratification Using a Novel Nomogram for 2190 EGFR-Mutant NSCLC Patients Receiving the First or Second Generation EGFR-TKI

John Wen-Cheng Chang, Chen-Yang Huang, Yueh-Fu Fang, Ching-Fu Chang, Cheng-Ta Yang, Chih-Hsi Scott Kuo, Ping-Chih Hsu, Chiao-En Wu

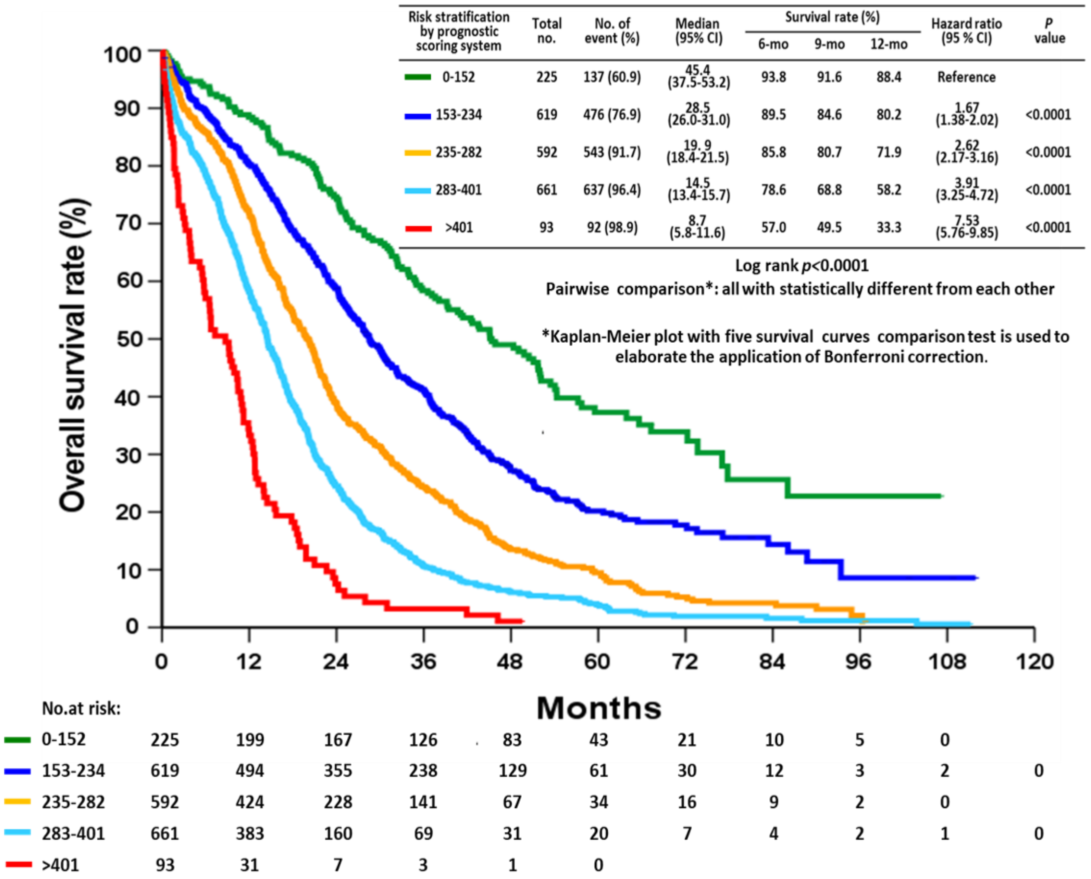

Figure S1. Kaplan-Meier plot of overall survival for 2190 patients according to risk stratification based on nomogram points.

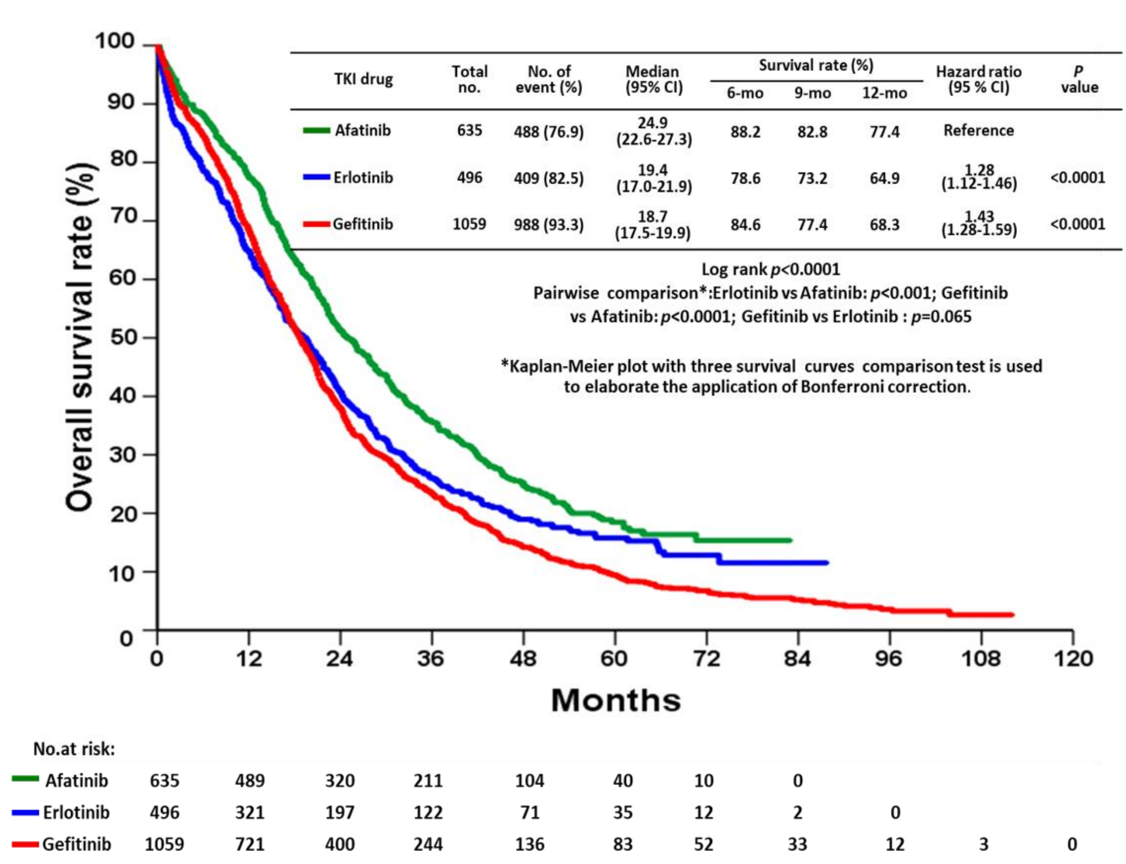

Figure S2. Kaplan-Meier plot of overall survival for 2190 patients according to the use of different EGKI-TKIs.

© 2022 by the authors. Licensee MDPI, Basel, Switzerland. This article is an open access article distributed under the terms and conditions of the Creative Commons Attribution (CC BY) license (http://creativecommons.org/licenses/by/4.0/).
